# Usefulness of Computed Tomography Imaging in Diagnosing Abdominal Compartment Syndrome: A Case Study

**DOI:** 10.7759/cureus.88788

**Published:** 2025-07-26

**Authors:** Miwako Hirota, Kyohei Nakade, Sakiko Masumoto, Nobuhiko Ogawa, Masaaki Tanaka

**Affiliations:** 1 Obstetrics and Gynecology, Fukui Prefectural Hospital, Fukui, JPN; 2 Radiology, Fukui Prefectural Hospital, Fukui, JPN

**Keywords:** abdominal compartment syndrome (acs), cardiopulmonary arrest, computed tomography (ct ), hypertensive disorders of pregnancy (hdp), obstetrical disseminated intravascular coagulation (dic), radiological diagnosis

## Abstract

We report a case of a patient who developed abdominal compartment syndrome (ACS) after cesarean section, leading to cardiopulmonary arrest, but was saved without serious sequelae. The patient, 32 years old, gravida 1, para 0, developed hypertensive disorders of pregnancy (HDP) at 27 weeks' gestation, with fetal growth restriction and elevated liver enzymes, and underwent emergency cesarean section at 30 weeks' gestation. On postoperative day 1, persistent bleeding from the uterine cesarean section scar to the abdominal cavity was observed, and uterine artery embolization stopped the bleeding. On the second postoperative day, the patient experienced increased abdominal distention, leading to cardiopulmonary arrest shortly after computed tomography (CT) imaging. Based on the imaging findings, the patient was suspected to have ACS, and emergency abdominal decompression was performed. CT findings such as narrowing of the inferior vena cava, elevation of the diaphragm, and distention of the extra-abdominal venous system were useful in the diagnosis of ACS.

## Introduction

Abdominal compartment syndrome (ACS) is a condition in which elevated intra-abdominal pressure causes organ damage, generally due to massive bleeding from severe trauma [1]. Elevated intra-abdominal pressure causes ileus and renal failure, and the subsequent increase in intrathoracic pressure results in respiratory failure and decreased cardiac output [2]. Although rare in the field of obstetrics and gynecology, a few cases have been reported due to ovarian tumors or rupture [3], ovarian hyperstimulation syndrome [4], excessive amniotic fluid from inter-fetal transfusion syndrome [5], and intra-abdominal bleeding after cesarean section [6]. We experienced a case of ACS after cesarean section due to intra-abdominal bleeding that resulted in cardiopulmonary arrest, but the patient survived without serious sequelae. A computed tomography (CT) scan was performed immediately before the cardiopulmonary arrest, and we discuss the CT imaging characteristics of ACS, which are different from those of hemorrhagic shock.

## Case presentation

The patient was 32 years old, gravida 1, para 0, with a spontaneous pregnancy. Her pre-pregnancy blood pressure was 130/100 mmHg. Systolic blood pressure increased to 170 mmHg at about 27 weeks' gestation, and fetal growth retardation of -2.8 SD was observed at 29 weeks. The patient was transferred to our hospital at 30 weeks 0 days. There was no medical history of note. Her mother had a history of hypertension. The patient was obese, with a height of 155 cm, a pre-pregnancy weight of 75 kg, and a BMI of 31.

On arrival (30 weeks 0 days), blood pressure was 159/101 mmHg, and the patient was started on the oral medication nifedipine 20 mg/day. The estimated fetal weight (EFW) was 953 g (-2.8 SD), amniotic fluid index 8.0 cm, umbilical artery resistance index 0.77, with no regurgitation or interruption of the umbilical artery, and the non-stress test showed a reassuring pattern. Blood tests showed mild renal dysfunction, with aspartate aminotransferase (AST) 22 U/L, alanine aminotransferase (ALT) 15 U/L, lactate dehydrogenase (LDH) 393 U/L, platelets 150,000/L, and creatinine 0.88 mg/dL (Table 1), and she was hospitalized and managed with a diagnosis of hypertensive disorders of pregnancy (HDP). At 30 weeks 4 days' gestation, blood pressure was 167/114 mmHg, she complained of headache, and blood tests showed elevated liver enzymes and decreased platelets, with AST 54 U/L, ALT 67 U/L, LDH 442 U/L, and platelets 110,000/L (Table 1). The EFW was 961 g (-3.0 SD), and no growth was observed. An emergency cesarean section was performed with a diagnosis of exacerbation of HDP and growth restriction. The operation time was 59 minutes, and blood loss was 450 g, including amniotic fluid. Uterine contractions were good, and after confirming no bleeding at the cesarean section scar, the operation was completed. The baby was born at 882 g (-3.0 SD), with an Apgar score of 3 at 1 minute and 7 at 5 minutes, and was managed in the Neonatal Intensive Care Unit.

**Table 1:** Blood test results before delivery AST: aspartate aminotransferase, ALT: alanine aminotransferase, LD: lactate dehydrogenase, Plt: platelet, Cre: creatinine.

Variables	AST (U/L)	ALT (U/L)	LD (U/L)	Plt (/L)	Cre (mg/dL)
Normal range	13-30	7-23	124-222	158,000-348,000	0.46-0.79
On admission	22	15	393	150,000	0.88
At 30 weeks 4 days' gestation	54	67	442	110,000	0.86

After returning to the room, the patient had transvaginal bleeding of approximately 1200 g in the first 7 hours postoperatively. Blood tests showed abnormal coagulation: hemoglobin (Hb) 9.3 g/dL, platelets 92,000/L, fibrinogen 160 mg/dL, and fibrin degradation products (FDP) 29.6 μg/mL (Table 2). Uterine contraction agents were administered, intrauterine balloon tamponade was performed, and blood was transfused with 4 units of fresh frozen plasma (FFP) and 10 units of platelet concentrate (PC). The next morning, a blood test showed marked progression of anemia, with Hb 5.1 g/dL, and contrast-enhanced CT showed leakage of contrast medium from the cesarean section scar, which was suspected to be bleeding into the abdominal cavity. Blood tests showed abnormal coagulation: platelets 139,000/L, fibrinogen 166 mg/dL, FDP 11.5 μg/mL, and antithrombin 3 (AT-3) 58% (Table 2). Transfusion of 10 units of FFP, 10 units of PC, and 8 units of red blood cells (RBC), and uterine artery embolization (UAE) was performed, and cessation of bleeding was confirmed on imaging. We did not choose relaparotomy because of abnormal coagulation.

**Table 2:** Blood test results after delivery Hb: hemoglobin, Plt: platelet, Fib: fibrinogen, FDP: fibrin degradation products, AT-3: anti thrombin 3.

Variables	Hb (g/dL)	Plt (/L)	Fib (mg/dL)	FDP (μg/dL)	AT-3 (%)
Normal range	11.6-14.8	158,000-348,000	172-383	0-10	80-120
7 hours after cesarean section	9.3	92,000	160	29.6	
The next morning	5.1	139,000	166	11.5	58

**Figure 1:**
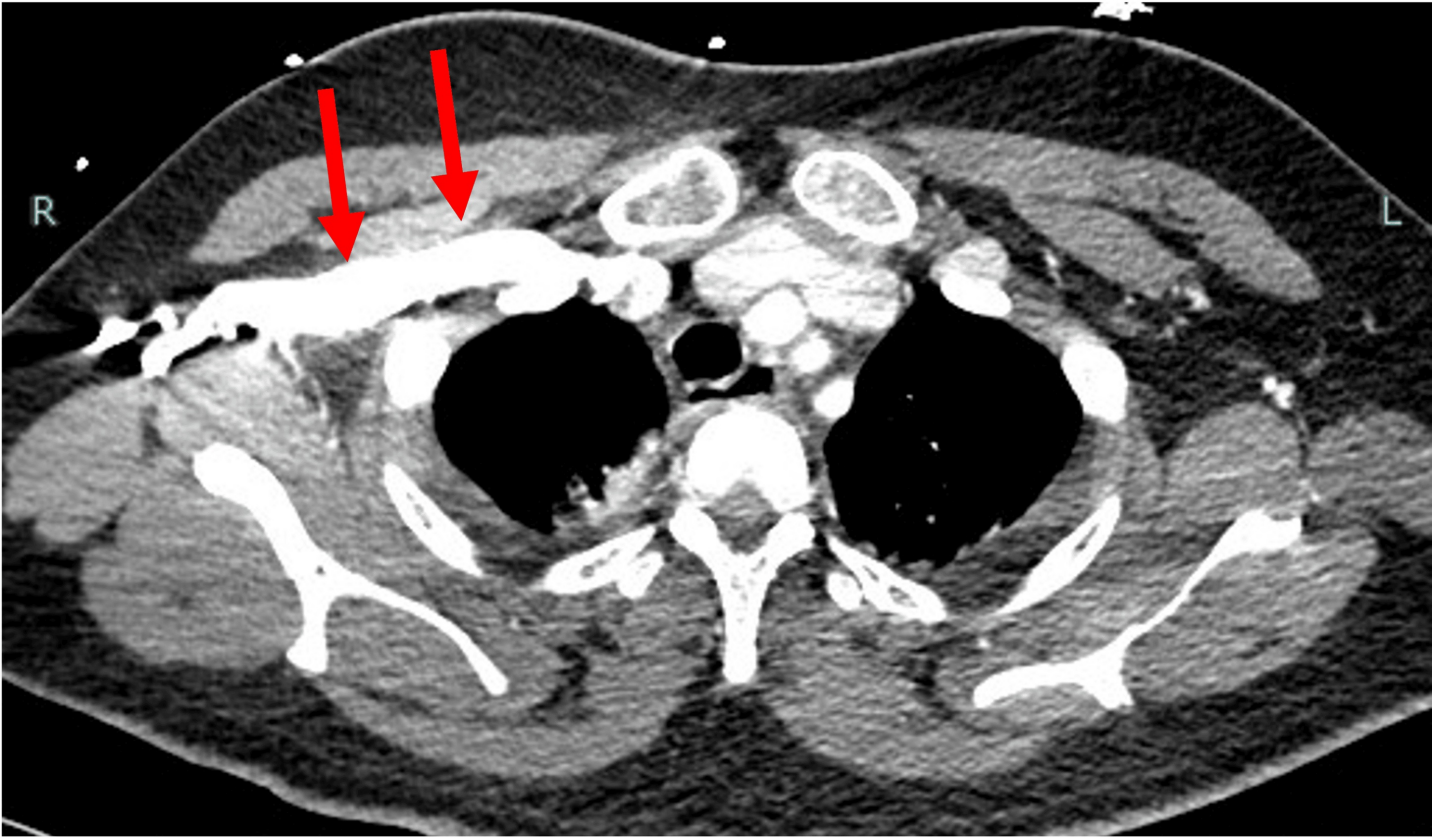
CT image at cardiopulmonary arrest. Contrast is highly stagnant in the brachiocephalic vein.

**Figure 2:**
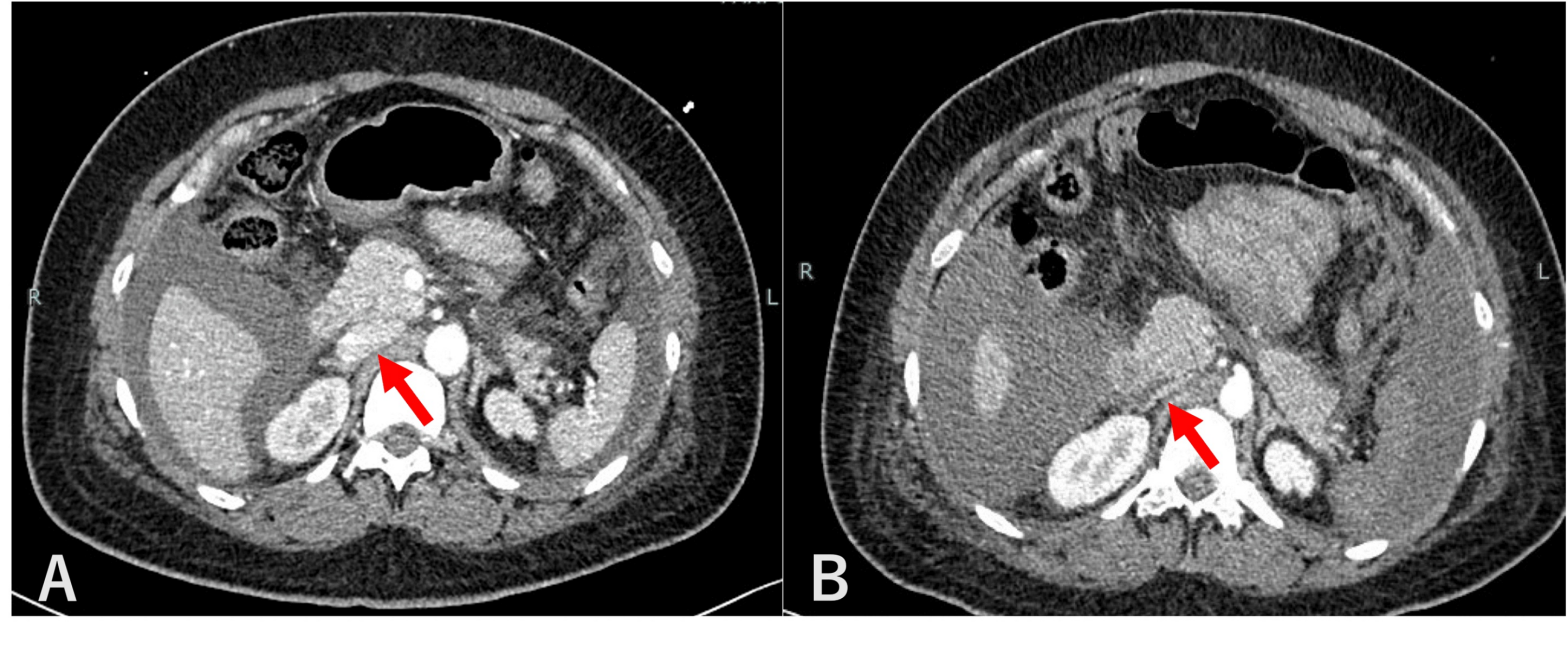
Comparison CT images of the inferior vena cava. (A) Image of the day before. (B) Image at cardiopulmonary arrest.

**Figure 3:**
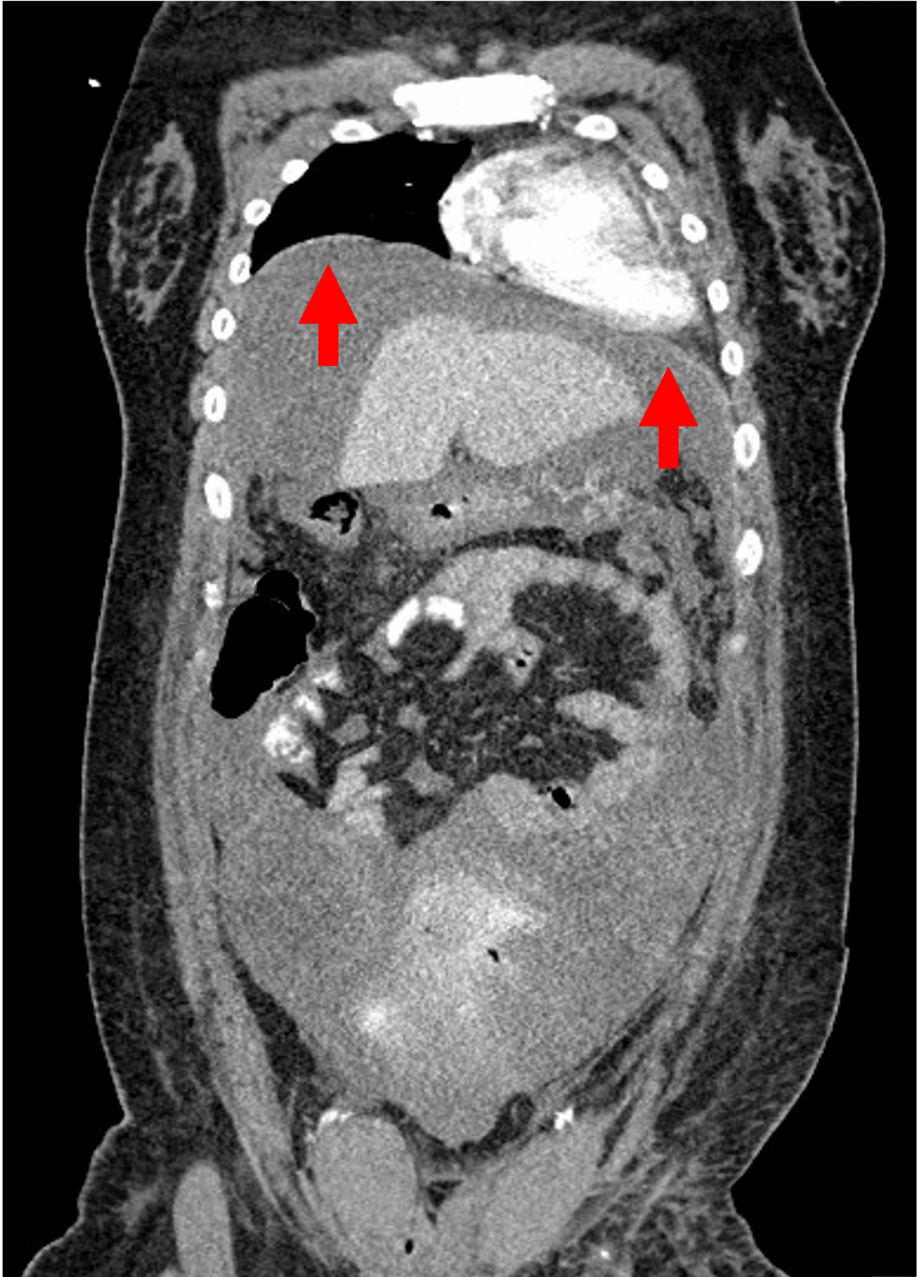
CT image at cardiopulmonary arrest. The dome of the diaphragm was elevated with the 5th thoracic vertebral body.

Thereafter, there was little transvaginal bleeding and blood pressure remained stable at around 110/70 mmHg, but 12 hours after UAE, the patient gradually began to complain of abdominal distention and dyspnea. At this time, her blood pressure was 99/65 mmHg and her heart rate was 115 beats/min. Compared to the previous day, Hb had decreased by 0.7 g/dL despite an additional transfusion of four units of RBC. Another contrast-enhanced CT scan showed no contrast leakage from the uterine incision, but bloody ascites had increased. Contrast was administered from a peripheral vein in the right upper extremity, and contrast was highly stagnant in the brachiocephalic vein to the right upper extremity vein (Figure 1). The inferior vena cava was highly narrowed with a short diameter of 1.9 mm (Figure 2), while the superior vena cava, internal jugular vein, and femoral vein were distended with diameters of 20 mm, 16 mm, and 10 mm, respectively. The dome of the diaphragm was elevated to the level of the fifth thoracic vertebral body (Figure 3). The ratio of anteroposterior to transverse abdominal diameter was 0.88, and the ratio of peritoneal to abdominal height was 0.61. The patient went into cardiopulmonary arrest immediately after the CT scan and was resuscitated 2 minutes later. Since ACS was suspected based on CT imaging findings, an emergency laparotomy decompression was performed. A total of 2800 g of bloody ascites and clots was stored in the abdominal cavity, and these were removed. There was no bleeding from the cesarean section scar, and the operation was completed with the placement of a drain. The postoperative course was good. For one week postoperatively, ascites drainage continued to be around 1000 mL per day. The patient was discharged home on the 15th day after cesarean section and the 13th day after cardiopulmonary arrest. There were no neurological sequelae, and both mother and child are doing well.

## Discussion

ACS is defined as multiple organ dysfunction caused by high intra-abdominal pressure (intra-abdominal pressure > 20 mmHg) [1]. Increased intra-abdominal pressure compresses the renal veins, resulting in decreased renal function and anuria; blocked blood flow to the intestinal tract results in intestinal edema and ischemia; and increased intrathoracic pressure causes decreased cardiac output and respiratory function [2]. If left untreated, it can lead to death from multiple organ failure. Symptoms may include fatigue, weakness, dizziness, dyspnea, abdominal distention, and abdominal pain.

How is ACS diagnosed? In principle, elevated intra-abdominal pressure must be confirmed. Measurement of intra-abdominal pressure is usually substituted by intravesical pressure [7]. Although intravesical pressure is relatively easy to measure, the sites where it is measured are often limited to intensive care units, and most physicians are unlikely to have experience in measuring intravesical pressure. Intravesical pressure is rarely measured in the maternity ward, where patients are rarely observed after trauma. In the absence of intra-abdominal pressure measurements, what tests are available to assist in the diagnosis of ACS?

Caruso et al. (2023) reported that ACS can be diagnosed using imaging findings on CT [8]. They outlined several imaging features indicative of ACS, which can be compared with the findings in the present case. In ACS, the inferior vena cava appears narrowed despite the absence of hypovolemic shock. In this case, the short diameter of the inferior vena cava was 1.9 mm (Figure 2), indicating narrowing (<3 mm). Diaphragmatic elevation was also listed as the most common finding. In this case, the dome of the diaphragm reached the level of the 5th thoracic vertebral body (Figure 3), thus meeting the definition of elevation (above the 10th thoracic vertebral body). The ratio of the anteroposterior to transverse abdominal diameter was 0.88 (>0.8), and the ratio of peritoneal to abdominal height was 0.61 (>0.52). The abdomen appeared distended, resembling a log covered with thin bark. Overall, half of the findings proposed by Caruso et al. were observed in this case.

In this case, cardiac arrest occurred immediately after CT imaging. The shock index before the CT scan was 1.1, suggesting the possibility of hemorrhagic shock. Severe narrowing of the inferior vena cava and elevation of the diaphragm (Figures 2-3) were observed, and these findings are consistent with a large amount of intra-abdominal bleeding. Although the extravascular leakage of contrast medium seen on the previous day had disappeared, the increased amount of bloody ascites compared to the CT of the previous day suggests that a small amount of bleeding persisted after UAE. However, despite the severe narrowing of the inferior vena cava, the superior vena cava, internal jugular vein, and femoral vein were all distended, and the contrast medium was highly stagnant in the brachiocephalic to right upper extremity veins (Figure 1). Based on the above, we considered that the severe narrowing of the inferior vena cava was not due to hemorrhagic shock, but to ACS.

In this case, the rapid progression of ACS resulted in cardiopulmonary arrest. What measures should have been taken to prevent this series of events? The patient presented with a condition suspicious for HELLP syndrome before the cesarean section, and since coagulation abnormalities are reported to occur in approximately 20% of patients with HELLP syndrome [9,10], postoperative coagulation abnormalities and subsequent intra-abdominal bleeding should have been considered. Mild organ failure was observed, and attention should also be paid to the possibility of generalized edema and ascites caused by vascular endothelial damage and renal dysfunction [11]. Postoperative ascites accumulation due to HDP should also be considered. If an intra-abdominal drain had been placed, postoperative intra-abdominal bleeding and ascites accumulation could have been monitored, and intra-abdominal decompression could have been performed. The patient also had risk factors for ACS, such as obesity and massive blood transfusions, and we should have been aware of her high risk for ACS [2].

## Conclusions

The patient developed ACS after cesarean section and went into cardiac arrest, but the imaging findings led to early treatment, and the patient was saved without serious sequelae. Narrowing of the inferior vena cava, elevation of the diaphragm, and distention of the extra-abdominal venous system were simple and useful findings on CT imaging to diagnose ACS. There is no reported case of cardiopulmonary arrest from ACS after cesarean section, and we believe that the CT imaging findings in this case are useful as diagnostic indicators of severe ACS.
